# Enhanced structural variant and breakpoint detection using SVMerge by integration of multiple detection methods and local assembly

**DOI:** 10.1186/gb-2010-11-12-r128

**Published:** 2010-12-31

**Authors:** Kim Wong, Thomas M Keane, James Stalker, David J Adams

**Affiliations:** 1 Wellcome Trust Sanger Institute, Hinxton, Cambridge, CB10 1SA, UK

## Abstract

We present a pipeline, SVMerge, to detect structural variants by integrating calls from several existing structural variant callers, which are then validated and the breakpoints refined using local *de novo *assembly. SVMerge is modular and extensible, allowing new callers to be incorporated as they become available. We applied SVMerge to the analysis of a HapMap trio, demonstrating enhanced structural variant detection, breakpoint refinement, and a lower false discovery rate. SVMerge can be downloaded from http://svmerge.sourceforge.net.

## Rationale

Next-generation sequencing technologies promise a revolution in our ability to understand the architecture of the human genome, and to decipher how this architecture contributes to disease [1]. This understanding is dependent on our ability to accurately detect differences between individuals on a genome-wide scale. Although nucleotide level variants such as SNPs and insertions/deletions (indels) are numerous, large structural variants, such as deletions, duplications and inversions, affect more sequence, and as much as 15% of the human genome falls into copy number variable regions [1]. Many of the software packages currently available to detect structural variants (SVs) employ algorithms that utilize data derived from the mapping of paired-end sequence reads, using anomalously mapped read pairs as a means for detecting and cataloguing these variants. Deletions, for example, are detected when the distance between mapped paired-end reads is significantly smaller than the average size distribution of other mapped read pairs from the same mate-pair sequencing library. Similarly, inversions may be identified when read pairs are mapped to the same strand of the reference genome. Examples of software using this approach include BreakDancer [2] and VariationHunter [3]. Other software packages such as Pindel [4] apply a split-mapping approach where one end of a pair of sequence reads is mapped uniquely to the genome and acts as an anchor, while the other end is mapped so as to detect the SV breakpoint. A third approach used to detect SVs involves ascertaining changes in read depth coverage, which reflect gains and losses in sequence copy number. Calling variants in this way will report regions of the reference genome that appear to be duplicated or deleted. This analysis, however, will not report the precise location of the duplicated sequence. Several algorithms have been developed for calling copy number variants in this way, including cnD, which applies a hidden Markov model to detect copy number variants [5], and RDXplorer, which uses a novel algorithm based on significance testing [6].

The location of large insertions can also be identified from mapping of paired-end sequence reads, where one end read is mapped to the reference sequence and the other end is either unmapped (for example, a novel sequence insertion), or mapped to another copy of the particular repeat element present in the reference (for example, insertion of a repetitive element, such as LINEs). We have developed two in-house tools, SECluster and RetroSeq[7], to detect these insertion events (see Materials and methods).

Independently, each of these approaches has limitations in terms of the type and size of SVs that they are able to detect, and no single SV caller is able to detect the full range of structural variants. The approach of utilizing paired-end mapping information, for example, cannot detect SVs where the read pairs do not flank the SV breakpoints, which can occur due to sequence features such as SNPs near the SV breakpoint, or where the number of supporting read pairs is low. Furthermore, the size of insertions that can be identified by paired-end analysis is limited by the library insert size. Insertion calls made using the split-mapping approach are also size-limited because the whole insertion breakpoint must be contained within a read. Read-depth approaches can identify copy number changes without the need for read-pair support, but cannot find copy number neutral events such as inversions, and read depth alone cannot be used to indicate the exact location of the duplicated sequence. For these reasons we developed SVMerge, a meta SV calling pipeline, which makes SV predictions with a collection of SV callers that are then merged, and computationally validated using local *de novo *assembly to gain a more comprehensive picture of the structural variants found within a genome. We show that SVMerge generates a more complete set of SV calls (>100 bp) compared to any single method alone, and provides refined SV breakpoints for downstream analysis. We have designed SVMerge to be both modular and extensible so that new SV calling methods may be incorporated into the analysis pipeline. Here we describe the main components of the pipeline, and results obtained from the analysis of three genomes from a HapMap trio sequenced using the Illumina platform. SVMerge is written in Perl and is freely available from [8].

## Results

### The SVMerge pipeline

**Figure 1:**
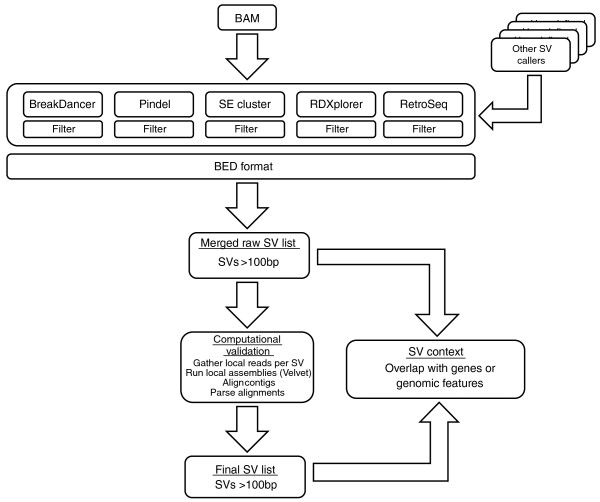
**An overview of the SVMerge pipeline**. SVMerge uses a suite of software tools to detect structural variants (SVs) from mapped reads. The calls are filtered, merged and then validated computationally by local *de novo *assembly. The output is in BED format, allowing for easy downstream analysis or viewing in a genome browser. The SVMerge pipeline is extendable so that calls made by other software can be included in the downstream analysis. BAM, Binary Alignment/Map format.

The SVMerge pipeline consists of four major modules: set-up and organization with a specific data structure, structural variant calling, filtering and merging of variant calls, and computational validation by *de novo *assembly using sequence reads mapped proximally to predicted breakpoints (Figure 1). The final output of SVMerge is in a standard Browser Extensible Data (BED) format, which greatly facilitates downstream analysis such as comparison of SV calls to gene lists using packages such as BEDTools [9], or visualization of SV calls using the UCSC [10] or Ensembl [11] genome browsers.

Each dataset to be analyzed is set up as a new project, and specific subdirectories are created to aid in the management of the data through each step of the pipeline. All configurable parameters, discussed below, are specified in a single configuration file that is used by SVMerge at each step of analysis as the pipeline is deployed. For each dataset to be analyzed, the user must supply either a single standard Binary Alignment/Map (BAM) formatted file with the reads aligned to a reference genome, or a set of BAMs consisting of one BAM per chromosome [12].

For the analysis presented here we have incorporated a variety of SV calling software packages into SVMerge. Table 1 lists the software, analysis method, and SV types called by these algorithms. The pipeline also allows for user-defined SV calls to be included, as the only requirement for subsequent steps of the pipeline is for SV calls to be in a specific tab-delimited format. Additional filtering, if required, is performed on the raw output of each SV caller to remove calls with weak support, or those calls that may be artifacts. This filtering includes the removal of calls of low quality, which is indicated by a score assigned to each SV prediction, and filtering of calls by their proximity to reference sequence assembly gaps, highly repetitive regions of the genome, and centromeres and telomeres. After filtering, the SV calls are separated by SV type, and calls of the same type from all SV callers are merged to generate a non-redundant set of calls (see Materials and methods). The raw, merged SV calls are output in tab-delimited format, indicating the chromosomal coordinates, and a unique identifier that provides information about the predicted SV type. This call set is used for subsequent steps in the SVMerge pipeline.

**Table 1:** Structural variation callers used in SVMerge

Software	Analysis method	SV types called	Size detection limitations
BDMax	Paired-end mapping	D, I, Inv, T	Insertions limited by library insert size
Pindel	Split-mapping	D, I, D+I	Insertions limited by read size; deletions <1 Mb
SECluster	Clusters of one-end-mapped reads	I	Minimum size dependent on insert size
RetroSeq	Targeted insertion calling	RI	Minimum size dependent on insert size
RDXplorer	Read depth	D, G	Minimum size approximately 1 kb

Listed are the software used to call structural variants (SVs), the analysis method used, SV types called, and limitations of the SV caller. 'BDMax' is BreakDancerMax. D, deletion; D+I, deletion with small insertion; G, copy number gain; I, insertion; Inv, inversion; RI, repeat insertion; T, translocation.

Following filtering and merging of SV calls, computational validation is performed using local *de novo *assembly of reads at predicted SV breakpoints, and comparison of the resulting contigs back to the reference genome. This serves to reduce the number of false positive SV calls, as well as aid in the refinement of SV breakpoints. This validation uses reads from the original BAM file mapped proximally to the predicted SV breakpoints. Because the accuracy of the SV boundaries in the raw, merged SV call set is dependent on which software is applied and the quality of the read mappings, the local assemblies are performed using SV boundaries that extend by at least 1 kb on either side (see Materials and methods). To simplify the process of potentially performing thousands of assemblies, SVMerge automatically generates configuration files, which specify all of the required parameters for the local assemblies and subsequent contig alignment steps. Currently, the assemblers supported by the pipeline are ABySS [13] and Velvet [14], but other assemblers can be incorporated by the user. ABySS and Velvet both provide an option to utilize scaffolding to join adjacent, non-overlapping contigs, which may be particularly useful when the reads used for a local assembly flank, but do not overlap, an SV breakpoint. Contigs generated from the local assembly step can be aligned to a reference chromosome, or to the slice of the reference to which the reads were mapped. The alignment tool used for this step of the pipeline is Exonerate, as the parameters of this mapper are easily configurable and the output format can be customized [15].

The final steps in SVMerge are automated parsing and interpretation of the contig alignment results to provide evidence supporting or refuting the original SV call. The first source of evidence is a breakpoint-containing contig, which either contains both ends of an SV breakpoint (for example, a contig contains an entire 300-bp insertion), or a single breakpoint (for example, half of a contig aligns to the reference and the other half is part of a larger insertion). For deletions, another source of evidence may simply be the lack of contigs covering the region predicted to be deleted (for example, homozygous deletions). Because heterozygous deletions may be difficult to validate by local assembly, an additional examination of mapped read depth is performed to ensure that heterozygous deletions are not falsely invalidated (see Materials and methods). For all SV calls with breakpoint-containing contigs, the alignment information is used to refine the original SV breakpoint predictions. We evaluated the accuracy of breakpoint localization obtained from the local assembly and contig alignment method using simulated data. Deletions, insertions, and inversions were added to the human chromosome 20 reference sequence, and reads were generated to produce a depth of coverage of 40 × (see Materials and methods). The breakpoint refinement step was able to determine the exact SV breakpoints for 60 to 89% of homozygous SVs, depending on SV type; this is significantly higher than the proportion from the raw output of the SV callers (Table 2). As expected, breakpoint refinement for heterozygous SVs was more difficult, especially for heterozygous insertions.

**Table 2:** Improvement of breakpoint resolution using local *de novo *assembly and breakpoint refinement in SVMerge

	Raw			Refined		
	
SV type	Called	Correct	Mean distance	Breakpoints detected	Correct	Mean distance
Homozygous						
Deletion (random)	99	9	+5/-3	99	77	-1/-1
Deletion (repeat)	99	4	+11/-8	99	89	0/0
Inversion	100	0	-169/175	85	46	51/24
Insertion	99	0	0/205	97	60	-1/1
Heterozygous						
Deletion (random)	96	2	+6/-4	96	40	-35/+18
Deletion (repeat)	94	0	+19/-15	91	35	0/2
Inversion	99	0	-166/+165	73	30	-58/+287
Insertion	96	0	+1/+202	18	18	0/0

To evaluate the performance of the local assembly and breakpoint refinement step in SVMerge, structural variants (SVs) were generated in human chromosome 20. For each category, 100 SVs were generated by random selection of location and size. Repeat deletions were selected from a list of LINEs and SINEs on chromosome 20. The raw, unfiltered calls are from BreakDancer raw output, except insertion calls, which are from SECluster raw output. 'Called' is the total number of SVs out of the 100 simulated SVs that were found in the raw output; 'Breakpoints detected' is the number of SVs, out of the total called, for which the SVMerge pipeline was able to detect breakpoints with the local assembly, and contig alignment and analysis steps; 'Correct' is the number of predictions that had matches to the actual breakpoint coordinates; 'Mean distance' is the mean distance from the actual breakpoints, where the numbers represent the 5'/3' breakpoints. The '+' indicates the mean distance was upstream of the actual breakpoint, and '-' indicates the mean distance was downstream. Raw and refined breakpoints were considered 'correct' if the direction and deviation at both the 5' and 3' breakpoints were equal.

Current SV callers do not attempt to call complex SVs, where more than one event appears to have taken place at a single locus. Local assembly analysis may reveal that a particular region is, in fact, complex, with more than one type of SV. SVMerge attempts to detect complex SVs, such as inversions that have occurred with a deletion or insertion, or a deletion that also contains an insertion at or near to the breakpoint. The final SV call set consists of deletions and copy number gains supported by read depth analysis, and complex SVs, deletions, insertions and inversions supported by local assembly analysis.

### Application of SVMerge to a HapMap trio dataset

We demonstrate the application of SVMerge to a dataset consisting of a high-depth HapMap trio (NA18506, NA18507, NA18508), which was sequenced on the Illumina platform [16]. The sequence data for these individuals were downloaded from the Sequence Read Archive (accession numbers [SRA009347], [SRA009225], [SRA000271]) and aligned to the GRCh37 human reference using BWA v0.5.5 [17] with default parameters. Sequence coverage depths for each genome were determined to be 42 ×, 42 × and 40 × for NA18506, NA18507, and NA18508, respectively. A single BAM file for each individual was produced for SV analysis (see Materials and methods).

The BAM file for each individual was used as input for BreakDancerMax [2], RDXplorer [6], SECluster (unpublished; see Materials and methods), and RetroSeq [7]; SVMerge provides a tool to read data from a BAM file to produce the necessary Pindel input file. Specific parameters for each SV caller are provided in the Materials and methods section. The resulting BreakDancerMax and Pindel calls were filtered, allowing a minimum score of 25. All SV calls were filtered by location, and calls less than 600 bp from a reference sequence assembly gap and 1 Mb from a centromere or telomere were excluded from further analysis. Only calls greater than 100 bp were considered; for RDXplorer the minimum size of SV calls included was 10 kb. BreakDancerMax is able to call inter-chromosomal translocations, although even with stringent filtering by score the number of translocation calls in these datasets was high (data not shown); since the majority of these are not likely to be real, they were excluded from further analysis. Table 3 shows the number and type of SV calls produced for the child, NA18506. Similar results were obtained for the parents (Additional file 1). A clear advantage was obtained by using a variety of SV calling methods, as demonstrated by comparing both the number of candidate calls and SV types for individual callers versus the 'merged raw' call set. Although read-pair analysis is able to find a large number of candidate deletions, deletions with low or no read-pair support require different approaches, such as split-read and read-depth analyses, which are provided by Pindel and RDXplorer, respectively. Similarly, the nominal overlap between the insertion calls from the different SV callers reflects the ability of the various callers to find insertions of a specific size or type.

**Table 3:** Structural variant calls for individual NA18506

Call set	Deletion	Insertion	Inversion	CNG	**Complex**^**a**^	Total
BDMax	4,141	1,844	324	-	-	6,309
Pindel	458	0	-	-	-	458
SECluster	-	1,215	-	-	-	1,215
RetroSeq	-	2,297	-	-	-	2,297
RDXplorer	575	-	-	280	-	855
Merged raw	4,717	5,252	324	280	-	10,573
SVMerge final	4,184	575	38	280	99	5,176

^a^'Complex' refers to any locus with more than one structural variant type - for example, an inversion with a deletion. Shown are the numbers of raw calls (>100 bp) from each structural variation (SV) caller, filtered by score and location only (see Materials and methods), for NA18506, the child in the HapMap trio dataset. 'BDMax' is BreakDancerMax. Pindel is able to identify deletions that also contain small insertions; these are included in the total deletion count. 'Merged raw' is the resulting number of calls after merging of these calls by their coordinates (see Materials and methods). 'SVMerge final' is the total number of calls made after refinement of the SV call list by local assembly and read depth analysis. Copy number gains (CNG) are not subject to validation by local assembly.

The 'merged raw' SV calls, excluding copy number gains and deletions without supporting read pairs or split-reads, were subjected to computational validation by local assembly. The 'final' calls for the child (NA18506) are shown in Table 3. Similar results were obtained for the parents (Additional file 1). The final call set contains all copy number gain calls and deletion calls unique to RDXplorer, insertions and inversions with evidence of breakpoints from local assembly analysis, and deletions with either supporting evidence from local assembly analysis or read-depth analysis (see Materials and methods). A number of complex SVs (for example, inversions with internal deletions) were also identified. Local assembly analysis was able to provide refined coordinates for approximately 60% of deletions in the 'final' call set. For the remaining deletions, where breakpoint refinement failed but read-depth analysis provided evidence of low or no read coverage, the original breakpoint coordinates from the 'merged raw' call set were used in the 'final' call set. Only 11% of insertions in the 'merged raw' call set were validated by local assembly and included in the 'final' call set, reflecting both the difficulty in detecting true insertions, as well as the difficulty validating these calls by local assembly.

Table 4 shows the number of SV calls in the 'final' call set that are unique to a specific SV caller, and those shared by at least one other caller. These numbers illustrate a clear benefit of complementing paired-end mapping analysis with read-depth analysis and other methods that target specific classes of repetitive elements. BreakDancerMax alone produced 3,874 of the 5,176 calls to the 'final' call set; the addition of the other callers provided an additional 1,302 calls that would have otherwise been undetected.

**Table 4:** Contribution of individual structural variant callers to the 'SVMerge final' call set for NA18506

	Unique SV calls					
			
	Deletion	Insertion	Inversion	CNG	Shared SV calls	Total SVs
BDMax	3,283	45	124	-	442	3,874
Pindel	25	0	-	-	404	429
SECluster	-	449	-	-	40	489
RetroSeq	-	44	-	-	7	51
RDXplorer	526	-	-	280	49	855

Shown are the number of structural variant (SV) calls (>100 bp) from each SV caller that are included in the 'final' call set for NA18506. 'Unique SV calls' are those that are made by a single SV caller only, and 'Shared SV calls' are SVs that were found by more than one method. 'BDMax' is BreakDancerMax. CNG, copy number gain.

To evaluate the final SV call set from SVMerge for the HapMap trio, we compared the overlap of the deletion, gain, and inversion calls against the curated Database of Genomic Variants (DGV; March 2010 release) [18]. SVMerge calls overlapped with calls in DGV at a rate significantly higher than expected by random chance (*P *< 0.001; Additional file 2). Of the deletion calls made by SVMerge, 71% (NA18506), 81% (NA18507), and 71% (NA18508) had a minimum 50% reciprocal overlap with regions of known copy number variation in DGV, while 29% (NA18506), 32% (NA18507), and 36% (NA18508) of the copy number gains overlap entries in DGV. The proportion of SVMerge inversion calls overlapping with known inversions in DGV was 47% (NA18506), 69% (NA18507), and 51% (NA18508). We further evaluated the calls in the child that did not overlap DGV calls by determining their presence in either parent's raw SV call set. This accounted for a further 18% of deletions, 32% of inversions, and 54% of duplications in the child to give us estimated maximum false discovery rates of 11%, 21%, and 17% for deletions, inversions, and copy number gains, respectively. Child-only insertions, with coordinates greater than 200 bp from any parental insertion, account for 14.4% of insertion calls. Collectively, all child-only SV calls comprise 11% of the child's final SV call set. This is a considerable improvement from what was observed in the 'merged raw' SV call set, in which 50% of the calls are unique to the child. The majority of these are false insertion calls from read pair analysis, which may be due to an artifact of the library construction; there is a significant tail at the lower end of the library insert size distribution (data not shown), which generates read pairs that align closer than expected based on the mean and standard deviation of the distribution. The maximum false negative rates, which can be estimated by assuming all of the child SV calls are real and comparing them to parental SV calls, are 21% for deletions, 40% for inversions, 23% for duplications, and 25% for insertions. False positive rates for individual structural variant callers and a comparison of confidence scores for child-only calls versus shared calls are provided in Additional files 3 and 4, respectively.

## Discussion

Genomic structural variation is increasingly being recognized as an important source of phenotypic variation [19]. The advent of new sequencing technologies means that it is now possible to create high-resolution maps of these variants; however, the range of SVs detected by individual SV callers is somewhat limited. For this reason, we have developed SVMerge, the first meta SV caller that integrates calls made from multiple sources and validates these calls using local *de novo *assembly. We illustrate that this approach produces a more comprehensive set of variant calls, compared to calls made by any single caller alone. SVMerge is an extensible pipeline that allows calls from any method to be easily incorporated. As more algorithms and a wider variety of SV callers become available, we expect that our complementary method will be able to produce an even wider spectrum of calls.

A key part of the SVMerge pipeline is the computational validation step, which performs local assembly and breakpoint refinement. Analysis of the HapMap trio has enabled us to demonstrate that this part of the pipeline can significantly reduce the false discovery rate. With simulated data, we show that this step also increases the accuracy of the predicted SV breakpoints compared to those produced by the individual callers (Table 2). However, computational validation of insertions and heterozygous SVs remains challenging. The proportion of computationally validated insertions is notably lower than other SV types. Local assembly of a *de novo *insertion is hampered by the lack of reads, since the reads provided for assembly are those that map near the breakpoint, or unmapped reads with mates that align near the breakpoint. Heterozygous breakpoint detection and refinement would be improved by advancements in methods such as short read assemblers that can perform diploid assemblies, or the use of assembly graphs to detect breakpoints. In the case of copy number gain and loss calls based only on read depth, local assembly is not a suitable validation strategy. As an alternative strategy, a high-confidence set of copy number gain and loss calls could be derived by applying more than one read-depth-based copy number caller and considering the intersection of calls.

Identifying SVs from short read data has its limitations, in particular with complex SVs where multiple rearrangement events occur at a single locus. SVMerge attempts to identify a subset of complex SVs by interpreting alignments of contigs generated from local assembly. However, these complex SVs are initially identified from read pair analysis only as single SV type. Improvements to SV calling algorithms, which can interpret complex read pair patterns, and the development of new long range sequencing techniques, such as strobe sequencing [20], will enable identification and elucidation of complex SVs that are currently difficult to characterize.

## Materials and methods

### Data

Sequence data for individuals NA18506, NA18507 and NA18508 were downloaded from the Sequence Read Archive (accession numbers [SRA009347], [SRA009225], [SRA000271]) and aligned to the GRCh37 human genome reference sequence using BWA v0.5.5 [17] with default parameters. After alignment, the quality values were recalibrated using software tools from GATK [21], merged to the library level where duplicates were removed with Picard [22] and then a single BAM file for each individual was produced.

### Structural variant callers

BreakDancerMax is included in the BreakDancer software package. The results described here were generated using BreakDancer-0.0.1r89 [23]. Default BreakDancerMax parameters were used, except a minimum mapping quality score of 20 and the minimum thresholds (expressed in standard deviations from the mean) for distance between mate pairs were set to 8, 8, and 7 for NA18506, NA18507, NA18508, respectively. All BreakDancerMax results were further filtered by score and size (100 bp minimum) to remove low confidence calls. Insertions with a score of 35 or less, and deletions with scores 30 or less, were excluded from further analysis. For inversions, the minimum BreakDancerMax score accepted was 30 with supporting reads from at least 3 read pairs, and a minimal size of 100 bp required. Pindel was downloaded from [24]. The insert size parameter was set to 200 bp and calls with scores less than 25 were excluded from further analysis. Deletions and insertions of at least 100 bp in size were considered for further analysis. SECluster, an in-house tool, attempts to locate large insertions by identifying adjacent clusters of forward and reverse-strand reads with unmapped mates. A minimum of five supporting reads mapped to the same strand, with minimal mapping qualities of 20, were required to form a cluster. Insertion signatures were then identified by comparing the outer coordinates of the forward and reverse clusters of reads allowing up to a 100-bp separation or a 50-bp overlap between the clusters. Mobile element insertions were identified using RetroSeq [7], which looks for clusters of reads with one end mapping to the reference and the other mapping to a mobile element in Repbase [25], or clusters of reads with mates mapped to a canonical mobile element on a different chromosome on the reference genome. Alignments to Repbase were performed with SSAHA2 [26] with a minimum of 90% identify and hit length of 36 bp for a match. For read-depth-based copy number calling using RDXplorer, the default Z-score of 5 threshold was used, but we applied a minimum size cutoff of 10 kb. In addition to the above parameters and filtering criteria, calls in all call sets were excluded if they fell within 600 bp of a reference sequence assembly gap, or within 1 Mb of a centromere or telomere. We refer to this resulting filtered set as the 'raw' calls.

### Merging of raw calls

The raw call sets were merged by SV type and chromosomal coordinate to generate a non-redundant SV call set. For example, all deletion calls from BreakDancer, Pindel, and RDXplorer were compared; if the coordinate spans from a BreakDancer deletion call and Pindel deletion call overlapped, then the calls were merged. The deletion calls from RDXplorer were then compared to the merged BreakDancer and Pindel set to identify additional deletions not detected by either BreakDancer or Pindel. The rules applied for merging calls are outlined as follows: (1) if the overlap is less than 75 bp and the non-overlapping portion from each call is between 50 and 200 bp, take the outer coordinates of the union of the spans; (2) if the overlap is less than 75 bp and the non-overlapping portion from each call is ≥200 bp, do not merge the spans; (3) otherwise, merge the calls and use the intersect of the spans.

Insertions from BreakDancerMax, RetroSeq, and SECluster were compared and merged by taking the intersection of overlapping spans. Inversions and copy number gains were only called by a single SV caller. The resulting 'merged raw' set consists of deletions, insertion calls, inversions, and copy number gains.

### Local assemblies and contig alignments

All SV calls, with the exception of copy number gains and deletion calls without any read pair support, were evaluated with local assembly of reads mapped near predicted SV breakpoints. Mapped reads, and any unmapped mate-pairs, within 1 kb of a predicted insertion breakpoint, or 2 kb of all other SV types, were extracted from the BAM files, formatted to FASTA format with interleaved read pairs, and assembled by Velvet (v.0.7.53). For inversions spanning over 10 kb, reads mapping within 5 kb of the insertion breakpoint were used for assembly. If the number of reads exceeded the maximum (over 10,000 reads for insertions, 200,000 for all other variant types), the SV call was deemed to be an artifact and was excluded from further analysis. The Velvet parameters used for each individual are shown in Table 5. Each local assembly was run with and without Velvet's scaffolding option. All contigs generated for each SV call were aligned to the corresponding region in the reference genome using Exonerate (v.2.2.0) using the parameters described in Table 6.

**Table 5:** Velvet parameters for each individual

Sample	hash_length	ins_len	Exp_cov	cov_cutoff
NA18506	29	220	35	2
NA18507	27	200	35	2
NA18508	29	200	35	2

**Table 6:** Exonerate (v.2.2.0) parameters used for mapping *de novo *contigs back to the reference genome

Parameter	Value	Parameter	Value
model	affine:local	gappedextension	FALSE
bestn	50; 100 for inversions	joinrangeext	300
gapexend	-3	score	15
dnahspdropoff	10	dna submatrix values (inversions)	5 for match, -15 for mismatch
hspfilter	200		

### Final call set

For each SV call evaluated by local assembly, contig alignments were computationally parsed to determine if there was supporting evidence for the SV, and to localize the breakpoints of the SV. Because reads that map to the breakpoints of heterozygous deletions are a mixture of reads that match the reference and reads that cross a breakpoint, local assembly may not always generate a breakpoint-containing contig. Therefore, for deletions with supporting read pairs flanking the predicted breakpoints, but no breakpoints found in assembled contigs, the original BAM files were used to check the read depth coverage across the predicted region. The Samtools 'pileup' utility [12] is used to report the depth of coverage and mapping quality of each base in the region. Any base with a root mean square mapping quality less than 30 is considered repetitive. If the majority of bases are not repetitive and the mean depth of coverage across the region is less than 0.85 times the mean depth of coverage across the genome, the predicted deletion is retained in the 'final' call set and the coordinates from the raw, merged set are used. Short read aligners such as Maq [27] and BWA [17] place non-uniquely mapping read pairs randomly at one of all possible sites, meaning reads will be mapped even where LINEs, SINEs or other repetitive elements are absent in the query genome. Therefore, in regions where the root mean square <30 and the depth is lower than 1.2 times the mean depth of coverage across the whole genome, the original deletion call (which was supported by spanning read pairs) is retained in the final call set. The 'SVMerge final' call set, therefore, consists of all SVs with breakpoints verified by local assembly analysis (deletions, inversions, insertions, complex SVs), deletions passing the read depth check, and all copy number gains from RDXplorer that passed initial filtering.

### Simulation data set

To evaluate the breakpoint refinement step in SVMerge, we generated deletions, insertions, and inversions on the human reference chromosome 20, and simulated 38-bp paired short reads, with a mean insert size distribution of 220 bp. For each type of SV, 100 homozygous and 100 heterozygous SVs were generated by random selection of location and size, and the exact breakpoints were recorded. Sizes were selected from a size distribution of those found by SVMerge in the NA18506 genome. Insertion sizes were selected from the deletion size distribution. Repeat deletions were randomly selected from a list of RepeatMasker LINE and SINE elements >100 bp on chromosome 20 downloaded from the UCSC genome browser website [10]. SNPs and indels were added at a rate of 1 per 1,000 bp, and read errors were added at rate of 1 per 100 bp. Reads were generated to produce a depth of coverage of 40 ×. Reads were aligned with BWA, as described above in the 'Data' section, and a BAM file was generated. BreakDancerMax and SECluster were run as described above.

## SVMerge software availability and requirements

All published software used for SV calling is freely available, and must be installed prior to running SVMerge. SVMerge is written in Perl and is freely available from http://svmerge.sourceforge.net. Additional Perl modules are described in the SVMerge documentation and available from CPAN [28].

## Abbreviations

BAM: Binary Alignment/Map; bp: base pair; BED: Browser Extensible Data; DGV: Database of Genomic Variants; GATK: genome analysis toolkit; LINE: long interspersed nuclear element; SINE: short interspersed nuclear element; SNP: single nucleotide polymorphism; SV: structural variant.

## Competing interests

The authors declare that they have no competing interests.

## Authors' contributions

KW, TMK, JWS, and DJA conceived the ideas in the paper. KW and TMK implemented the software and carried out the data analysis. All authors read and approved the final manuscript.

## Supplementary Material

Additional file 1 **SV calls for individuals NA18507 and NA18508, made by individual SV callers and the final merged set**. Click here for file

Additional file 2 **Number of SVMerge final SV calls overlapping DGV entries for the trio, compared to random sets**. Click here for file

Additional file 3 **Comparison of the false discovery rates of individual SV callers**. Click here for file

Additional file 4 **Comparison of the confidence scores of SVs unique to the child and those shared with the parents**. Click here for file
