# Supplementary material for: Enhanced structural variant and breakpoint detection using SVMerge by integration of multiple detection methods and local assembly
Source: Genome Biol. 2010 Dec 31;11(12):R128. doi: 10.1186/gb-2010-11-12-r128 (PMC3046488; doi:10.1186/gb-2010-11-12-r128)
Supplement: Additional file 1 — SV calls for individuals NA18507 and NA18508, made by individual SV callers and the final merged set. [file gb-2010-11-12-r128-S1.doc]

**Additional File 1: Structural variant calls made from a collection of software tools, and final call set derived from SVMerge**.

|  | **Deletion** | | **Insertion** | | **Inversion** | | **CNG** | | **Complex** | |
| --- | --- | --- | --- | --- | --- | --- | --- | --- | --- | --- |
|  | NA18507 | NA18508 | NA18507 | NA18508 | NA18507 | NA18508 | NA18507 | NA18508 | NA18507 | NA18508 |
| **BDMax** | 3884 | 4128 | 1396 | 1917 | 272 | 308 | - | - | - | - |
| **Pindel** | 375 | 409 | - | - | - | - | - | - | - | - |
| **SECluster** | - | - | 2714 | 1043 | - | - | - | - | - | - |
| **RetroSeq** | - | - | 2240 | 1559 | - | - | - | - | - | - |
| **RDXplorer** | 428 | 588 | - | - | - | - | 239 | 238 | - | - |
| **Merged raw** | 4288 | 4700 | 6165 | 4501 | 272 | 308 | 239 | 238 | - | - |
| **SVMerge final** | 3803 | 3733 | 567 | 545 | 16 | 39 | 239 | 238 | 78 | 92 |

Here we show the number of raw structural variant (SV) calls from each SV caller, filtered by score and location only (see Methods). “BDMax” is BreakDancerMax. The numbers represent variants called from the parents of child NA18506 (father: NA18507 and mother: NA18508). 'Merged raw' is the total number of calls made after integrating the calls made by the five SV callers. 'SVMerge final' is the total number of calls made after refinement of the SV call list by local assembly and read depth analysis. Copy number gains (CNG), based on read depth alone, are not subject to validation by local assembly.
