# Supplementary material for: Enhanced structural variant and breakpoint detection using SVMerge by integration of multiple detection methods and local assembly
Source: Genome Biol. 2010 Dec 31;11(12):R128. doi: 10.1186/gb-2010-11-12-r128 (PMC3046488; doi:10.1186/gb-2010-11-12-r128)
Supplement: Additional file 2 — Number of SVMerge final SV calls overlapping DGV entries for the trio, compared to random sets. [file gb-2010-11-12-r128-S2.doc]

**Additional File 2: Comparison of final SVMerge calls with the Database of Genomic Variants**.

|  | Deletions | | | Copy number gain | | | Inversion | | |
| --- | --- | --- | --- | --- | --- | --- | --- | --- | --- |
|  | **Total** | **DGV** | **Random** | **Total** | **DGV** | **Random** | **Total** | **DGV** | **Random** |
| **NA18506** | 4184 | 2991 | 39 (20-65) | 280 | 82 | 11 (4-24) | 38 | 18 | 0 (0-1) |
| **NA18507** | 3803 | 3096 | 31 (18-51) | 239 | 76 | 10 (2-22) | 16 | 11 | 0 (0-1) |
| **NA18508** | 3733 | 2644 | 14 (4-25) | 238 | 86 | 10 (2-22) | 39 | 20 | 0 (0-1) |

Deletions, copy number gains, and inversions from each individual (“Total”) were compared to the Database of Genomic Variants (DGV). The deletions and copy number gains from SVMerge were compared to regions annotated as copy number gain/loss regions in the DGV, while the SVMerge inversions were only compared to known inversions in the DGV. The requirement for a match was a minimum 50% reciprocal overlap of the chromosomal coordinates. The number in the “DGV” column indicates the number of SVMerge calls matching a DGV call. For each SV type, we generated 1000 sets of calls with randomly chosen coordinates. These random sets each had same number of calls and same size distribution as the SVMerge call set. The numbers in the “Random” column are the mean number of calls from the random call sets that matched a DGV call. The minimum and maximum number of matches are shown in parentheses. In all cases, the number of SVMerge calls overlapping DGV calls was significantly higher than expected by random chance (p < 0.001).
