# Supplementary material for: Enhanced structural variant and breakpoint detection using SVMerge by integration of multiple detection methods and local assembly
Source: Genome Biol. 2010 Dec 31;11(12):R128. doi: 10.1186/gb-2010-11-12-r128 (PMC3046488; doi:10.1186/gb-2010-11-12-r128)
Supplement: Additional file 3 — Comparison of the false discovery rates of individual SV callers. [file gb-2010-11-12-r128-S3.doc]

**Additional File 3:** False positive rates of individual structural variant callers.

| SV Caller | SV Types | Raw calls | Not in DGV or Parents | False discovery rate (%) |
| --- | --- | --- | --- | --- |
| BDMax | Deletion, inversion | 4322 | 548 | 13.7 |
|  | Insertion | 173799 | 171603 | 98.7 |
| Pindel | Deletion | 543 | 50 | 9.2 |
| SECluster | Insertion | 1215 | 302 | 24.9 |
| RetroSeq | Insertion | 2279 | 1419 | 62.3 |
| RDXplorer | Deletion, copy number gain | 855 | 206 | 24.1 |

The structural variant (SV) caller raw output for the child, NA18606, was compared to the Database of Genomic Variants (DGV) and the raw output from the parents, NA18507 and NA18508. Since most of the child’s SV are expected to be found in either parent, we can estimate a false discovery rate the each caller. BreakDancerMax (BDMax) insertions were considered separately, since the majority of these calls are due to an artefact of the library insert size distribution. The requirement for a match was 50% reciprocal overlap, with the exception of insertions, which were considered the same event if they mapped the within 200bp of each other. A caveat to this method in estimating false discovery rates is that false SV calls due to mapping artefects may be consistent across data sets, and therefore falsely included as a true positive.
