# Supplementary material for: Enhanced structural variant and breakpoint detection using SVMerge by integration of multiple detection methods and local assembly
Source: Genome Biol. 2010 Dec 31;11(12):R128. doi: 10.1186/gb-2010-11-12-r128 (PMC3046488; doi:10.1186/gb-2010-11-12-r128)
Supplement: Additional file 4 — Comparison of the confidence scores of SVs unique to the child and those shared with the parents. [file gb-2010-11-12-r128-S4.doc]

**Additional File 4: Mean confidence scores for structural variant calls in the child (NA18506).**

|  | Raw | | Final | |
| --- | --- | --- | --- | --- |
| SV Caller | Unique to child | Shared with parent | Unique to child | Shared with parent |
| BDMax | 54 | 88 | 62 | 89 |
| Pindel | 36 | 46 | 36 | 46 |
| RDXplorer | N/A | N/A | 16 (gain); -5 (loss) | 94 (gain); -9 (loss) |

When compared to either the ‘raw’ structural variant (SV) calls from the parents (NA18507 and NA18508), or the ‘final’ parental SV calls, the calls unique to the child tend to have lower scores. BreakDancerMax (BDMax) and Pindel provide scores based on read supports for each call; RDXplorer provides a Z-score. The SV calls based on read depth (RDXplorer) are not subjected to local assembly validation and therefore are all included in the final call set.
